# The Design and Validation of a High-Precision Angular Vibration Calibration System Based on a Laser Vibrometer

**DOI:** 10.3390/s24196228

**Published:** 2024-09-26

**Authors:** Xinghan Lin, Zhigang Huang, Keyou Guo, Gang Li

**Affiliations:** School of Artificial Intelligence, Beijing Technology and Business University, Beijing 100048, China; xinghan_lin@sina.com (X.L.); guoky@th.btbu.edu.cn (K.G.); ligang@btbu.edu.cn (G.L.)

**Keywords:** high-precision angular vibration calibration system, angular vibration platform design, simulation analysis, accuracy testing

## Abstract

This paper presents the design and validation of a high-precision angular vibration calibration system based on a laser vibrometer, aimed at meeting the high-precision requirements for measuring small angular vibrations. The system primarily consists of a self-driving angular vibration platform and a laser vibrometer. The platform is isolated from ground interference via an air-floating platform and uses a split-type motor to control the platform, generating specific angular vibrations. Detailed simulations of the platform’s modal characteristics and the stability of the spring plates were conducted using the finite element analysis software ANSYS 11. Moreover, fundamental frequency testing and measurement accuracy testing were conducted on the system. Experimental results demonstrate that the system has a fundamental frequency of 2.69 Hz and a maximum measurement error of 0.00172″, confirming the system’s effectiveness in dynamic characteristics, stability, and measurement accuracy. This research provides essential technical support for high-precision angular vibration control in spacecraft.

## 1. Introduction

With the increasing frequency of human space activities such as interstellar exploration [1], space laser communication [2], space remote sensing [3], and space astronomical observation [4], the demand for pointing accuracy and the stability of spacecraft is increasing. The National Aeronautics and Space Administration (NASA) interstellar exploration program has proposed that spacecraft pointing accuracy and stability indicators should reach the sub-milliarcsecond level. The pointing accuracy of a long-baseline laser communication spacecraft in space needs to reach the microradian level [3,4,5,6]. The LISA, Taiji, and Tianqin programs for space gravitational wave detection require spacecraft inter-satellite pointing control accuracy to reach the order of 10 nanoradian [7]. The micro-vibrations of spacecraft can significantly impact the stability of vibration-sensitive payloads such as laser communication terminals and remote sensing cameras. By detecting and controlling these micro-vibrations, the performance of the spacecraft can be effectively enhanced [8].

Micro-vibration is a prevalent interference factor in spacecraft on orbit, attracting widespread attention due to its low amplitude and broad frequency distribution. The micro-vibration mainly originates from components such as flywheels, torque gyroscopes, and solar panels. Its spectrum covers a wide range of kHz, with the main energy concentrated in the low-frequency band below 100 Hz. The amplitude is typically less than 100 μrad and decreases with increasing frequency. This micro-vibration is characterized by periodicity or quasi-periodicity and is influenced by the design of the satellite structure; in particular, satellites with many payloads and which are light weight are more likely to produce large vibration amplitudes [9].

Research indicates that micro-vibration primarily affects the pointing accuracy and attitude stability of spacecraft, particularly causing significant interference with high-precision optical payload imaging, making it one of the main factors limiting spacecraft precision [10]. Different payloads have varying sensitivities to different degrees of micro-vibration, with optical payloads typically being more sensitive to angular vibrations than linear vibrations [9]. The control of micro-vibrations is a significant challenge in the development of high-precision, highly stable spacecraft [11]. Therefore, evaluating and controlling the impact of micro-vibration is crucial for ensuring the successful execution of spacecraft missions [12].

Various strategies have been developed to suppress the micro-vibration. Passive control technologies typically involve the use of vibration absorbers and isolation systems [13]. NASA has implemented a passive isolation platform in the Advanced X-ray Astrophysics Facility (AXAF) to isolate flywheels, using nonlinear springs with low stiffness near the balance point and increasing stiffness away from it [14], with experimental results demonstrating effective vibration isolation. The WorldView-2 satellite was equipped with four control moment gyroscopes (CMGs) using eight isolators [15] to reduce micro-vibrations, thus providing 0.5 m panchromatic and 1.8 m multispectral image resolution. Active control technologies utilize sensors and actuators to monitor and counteract vibrations in real time, offering more precise control across a broader frequency range. Kamesh [16] proposed an active elastic support vibration isolation device for flywheels This device consists of a flexible bracket vibration isolator with five folding beams for passive isolation, and piezoelectric patch actuators added on vertical support beams for active isolation based on optimal control theory. Due to the demands for the suppression of six-degrees-of-freedom vibration in space, the Stewart platform-style [17] active control is often applied for the vibration isolation of high-precision sensitive payloads. Semi-active control technologies adjust system parameters to adapt to varying vibration environments and also offer a more energy-efficient solution. Variable-frequency dynamic vibration absorbers introduce stiffness-adjustment mechanisms into the structure of passive dynamic vibration absorbers to maintain optimal damping effectiveness as the excitation frequency changes [18]. All the above research relies on a priori knowledge about the target vibration sources to better select parameters of the design and control method. Thus, precise prediction of micro-vibration can be vital.

The analysis and prediction of micro-vibration primarily rely on computational methods such as finite element analysis (FEA). D. Kamesh et al. [14] constructed a nonlinear dynamic analysis model of the reflector based on the characteristics of the laminated shell structure of a rigid–flexible coupled antenna. They analyzed its micro-vibration characteristics and proposed a PD (proportional-derivative) force controller to suppress the vibration of the antenna reflector. Wang et al. [19] used ANSYS 11 finite element software to analyze the fixed frequency and modes of a large flexible deployable antenna. Based on the modal resonance points, they designed a preloaded multi-lobe damping friction structure suitable for the antenna deployment mechanism. This structure effectively suppresses modal resonance points, and dynamic analysis of the antenna deployment process demonstrates the damping structure’s vibration reduction effect and feasibility. S. Miller [20] was the first to propose this semi-analytical model by building on the Masterson rotor dynamics model and adding axial degrees of freedom to the flywheel rotor. In this approach, both resonant disturbances and broadband disturbances are simultaneously introduced as inputs to the flywheel system. Structural dynamic derivation and modeling are then carried out. After the modeling is completed, disturbance tests on the flywheel system are conducted experimentally, and the analytical model is refined using the experimental data to obtain the final flywheel disturbance model. This model effectively combines the advantages of empirical and analytical models, not only reflecting the structural dynamic characteristics of the flywheel system but also accurately describing the broadband disturbances at low rotational speeds of the flywheel. However, these methods have certain limitations in precise simulation and isolation design, especially when dealing with complex real-world conditions. Consequently, ground-based micro-vibration testing has become an important supplementary method for validating design solutions, predicting on-orbit performance, and evaluating isolation system effectiveness. Ground tests not only provide experimental data but also aid in the development of more effective micro-vibration isolation technologies. By simulating the mechanical environment of the spacecraft in orbit on the ground, the micro-vibration characteristics of the spacecraft in orbit are evaluated to implement effective vibration suppression measures.

As a critical parameter for attitude measurement and control, the accuracy of angular vibration parameter evaluation is increasingly emphasized. The performance study of angular vibration tables, which serve as key devices for such evaluations, is gaining significant attention. Mathematical methods are insufficient to accurately evaluate angular vibration values [21,22], necessitating the dynamic calibration of sensors and angular vibration measurements. In standard angular vibration measurements, researchers have used tachometers and angle encoders, but the electromagnetic interference from tachometers is significant, making it difficult to meet measurement standards, and the angular subdivision precision of angle encoders do not satisfy the requirements for measuring high-frequency, low-amplitude angular vibrations. Some scholars have used corner cube prisms as the target for laser interferometry measurement schemes. However, at large angles, the Doppler frequency shift output from the interferometer does not have a linear relationship with the angle, and the installation of corner cube prisms occupies space on the surface of the angular vibration table, limiting their use in calibration applications. The German PTB has employed a laser interferometry measurement scheme using a ribbon diffraction grating as the cooperative target, although this approach presents significant manufacturing challenges [23].

To meet the research needs for measuring and controlling small angular vibrations, this paper designs a high-precision angular vibration platform to provide a stable experimental environment for small angular vibrations. The platform is designed for the calibration of high-sensitivity sensors, with a load capacity not exceeding 10 kg and an operating frequency range within 500 Hz, specifically for measuring rotation around the *z*-axis. This platform features self-driving capability and, in conjunction with a laser vibrometer, forms a high-precision angular vibration calibration system. To verify whether the measurement range and accuracy of the self-developed high-precision angular vibration calibration system meet the requirements, a high-precision laser gyroscope is used to test the accuracy of the calibration system.

## 2. Design and Simulation

### 2.1. Design of the High-Precision Angular Vibration Calibration System

**Figure 1:**
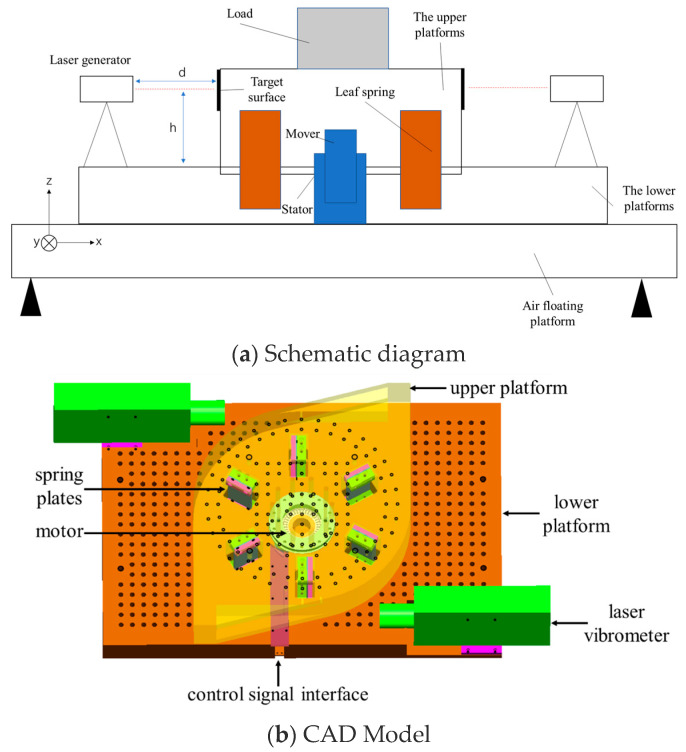
Schematic diagram of the high-precision angular vibration calibration system.

The high-precision angular vibration calibration system is composed of two main parts: the platform structure and the measurement equipment. The platform structure consists of an upper platform, a lower platform, a motor, and supporting spring plates, while the measurement equipment selected is a calibrated laser vibrometer, with a displacement resolution better than 15 p.m., meaning the error of the laser vibrometer is less than 15 p.m. The overall system model is shown in Figure 1.

For the platform structure, the upper platform has envelope dimensions of 600 mm × 480 mm × 90 mm and a mass of approximately 50 kg. It features threaded holes on the upper surface for mounting loads and a side target surface for reflecting the laser emitted by the laser generator. The lower platform has envelope dimensions of 880 mm × 600 mm × 65 mm, with a mass of about 78 kg. It is rigidly connected to the air-floating platform during operation to isolate ground interference; the air-floating platform isolates ground disturbances through air suspension, and since its fundamental frequency is very low, close to 0 Hz, it provides excellent vibration isolation effects. Additionally, it is equipped with two laser generators to measure the rotational angle between the upper and lower platforms.

The upper and lower platforms are connected by six spring plates. These plates provide sufficient support stiffness in the x, y, and z directions while maintaining low rotational stiffness around the *z*-axis of the upper platform. This design facilitates easy rotation around the *z*-axis between the upper and lower platforms. Additionally, different thicknesses of spring plates can be selected to adjust the system’s stiffness characteristics. Both platforms are equipped with a split-type rotating motor. The motor stator is fixed to the lower platform, while the rotor is attached to the upper platform. By outputting torque from the motor, the system can precisely control the upper platform to generate angular vibrations with specific frequencies and amplitudes. The turntable is designed for ease of use, and since its main purpose is to compare the measurement results between the two sensors, a simpler non-closed-loop control is adopted to ensure the stability of angular vibration through structural design.

### 2.2. Simulation Analysis of the High-Precision Angular Vibration Calibration System

This section performs a finite element analysis of the platform structure to verify whether its dynamic characteristics meet the usage requirements. Since the lower platform is connected to the base, this part can be simplified using fixed boundary conditions. Therefore, the simulation analysis mainly includes the following aspects: modal analysis of the upper platform, stability analysis of the spring plates, and modal analysis of the overall structure.

#### 2.2.1. Modal Analysis of the Upper Platform

**Figure 2:**
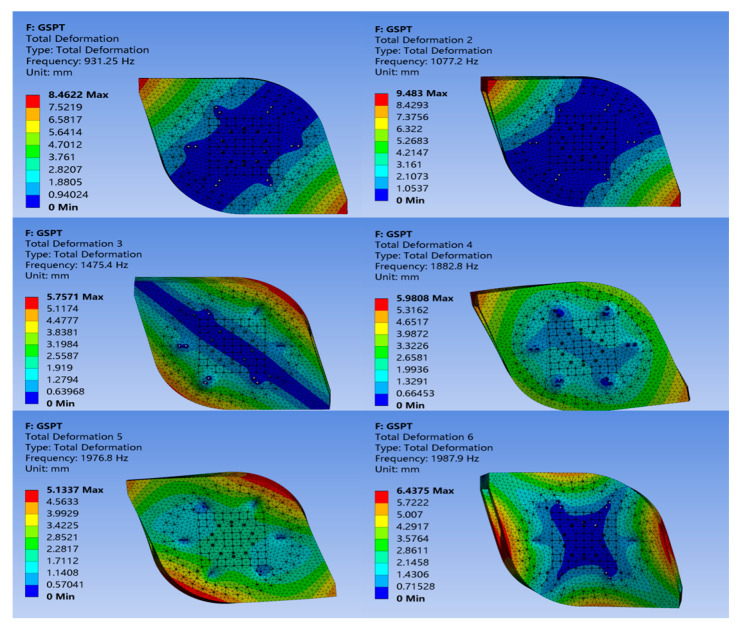
First six modal shapes of the upper platform.

In the ANSYS 11 finite element simulation software, modal analysis is first performed on the upper platform to extract the frequencies of various modes. After importing the model into ANSYS, fixed supports are assigned to the spring plate connection holes on the upper platform (6 locations, totaling 12 holes). Since local modes can influence the actual measurement results, a transfer function test based on a swept-frequency signal was conducted to avoid exciting local modes by bypassing the resonance peaks, so the impact hammer test was not used for modal analysis. After solving the simulation, the first six mode shapes of the upper platform are shown in Figure 2, with the modal frequencies listed in Table 1. The simulation results indicate that the fundamental frequency of the upper platform is 931.25 Hz.

#### 2.2.2. Stability Analysis of the Spring Plates

Due to the fact that the spring plates in the turntable structure are both wide and thin, they can be considered as thin-plate structures. Under in-plane pressure, thin plates may experience instability, leading to a decrease in the overall load-bearing capacity or abnormal deformation of the component. Therefore, it is necessary to analyze the compressive stability of the spring plates.

Given the boundary conditions, a single spring plate can be equivalently modeled as a cantilever plate under compressive stress. The pressure is equal to the sum of the gravitational force of the upper platform and the load and is directed parallel to the mid-plane of the spring plate. The critical stress $\sigma_{\mathrm{cr}}$ can be expressed as follows:

$$\sigma_{\mathrm{cr}}=\frac{\pi^{2}EK}{12\left(1-\nu^{2}\right)}\frac{t^{2}}{b^{2}}$$

where *K* represents the stability coefficient, taken as 1.28; ν denotes the Poisson’s ratio, and *E* denotes the Young’s modulus of the material. For the material 65 Mn, these parameters are 0.3 and 210 GPa, respectively; *b* is the width of the thin plate, set at 60 mm, and *t* is the thickness of the thin plate, which can be selected within the range of 0.6 mm to 3.5 mm.

To analyze the stress conditions of the spring plates, shell elements are used for finite element modeling of the spring plates. Fixed constraints are applied to the lower ends of the spring plates to simulate the fixed boundary conditions of the lower platform during operation. The upper platform is modeled as a mass point, connected to the upper end of the spring plate through a Multi-Point Constraint (MPC), and a vertical downward force is applied at the mass point.

**Figure 3:**
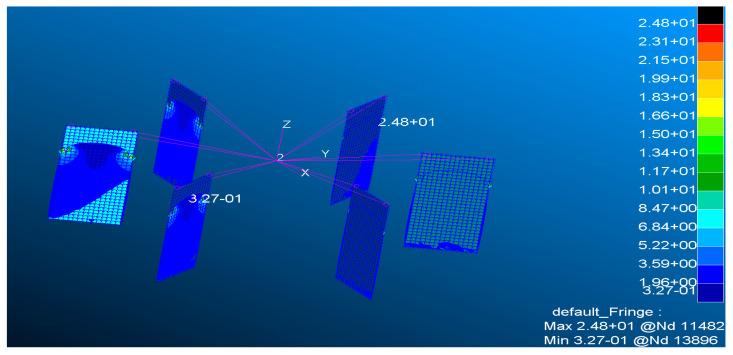
The stress analysis results of the spring plates.

For when a pressure of 600 N is applied, the stress analysis results for the 0.6 mm thick spring plate are shown in the Figure 3. The critical stress and maximum stress of the spring plates under different thickness conditions are listed in the Table 2. It can be observed that the spring plates with the specified thickness all meet the stability requirements, and there is no occurrence of instability in the support provided by the spring plates.

#### 2.2.3. Modal Analysis of the Overall Structure

To avoid interference from modes of vibration not related to the rotation of the upper platform, the first mode of the overall structure, where the lower platform is fixed and the upper and lower platforms are connected by a spring plate, should be the rotation of the upper platform, and this mode’s frequency should be significantly lower than that of the other modes. The first mode rotates around the *z*-axis. Using the same simplified model as in the spring plate stability analysis, a modal analysis is performed, with no applied force at the mass point in this analysis.

**Figure 4:**
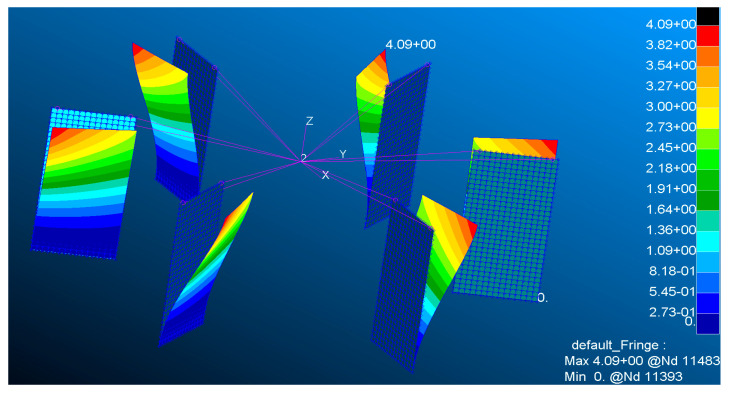
The first mode shape of the overall structure.

For when 0.6 mm thick spring plates are used, the first mode shape of the overall structure is shown in the Figure 4. For different thicknesses of spring plates, the first four modal frequencies of the overall structure are listed in the table below. As the simulation results in Table 1 show that the fundamental frequency of the upper platform is significantly higher than the expected frequency range, it is concluded that the results in Table 3 can effectively reflect the overall modal characteristics of the turntable, and the modal frequencies of the overall structure meet the usage requirements. Therefore, a spring sheet with a thickness of 0.6 mm was ultimately used for the actual test.

## 3. Measurement Accuracy Validation of the High-Precision Angular Vibration Calibration System

**Figure 5:**
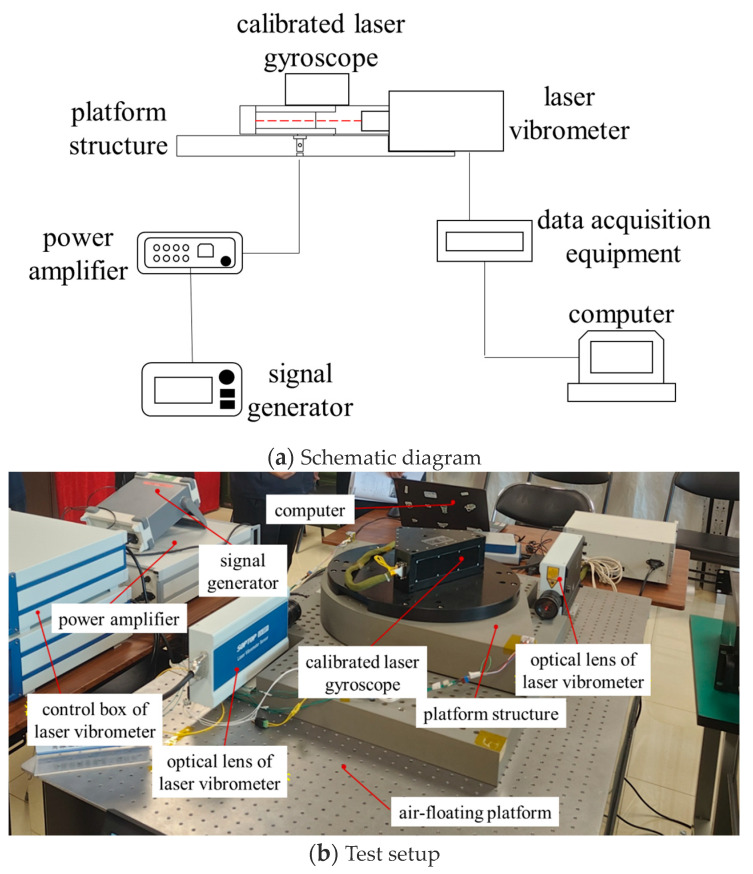
The measurement accuracy verification test of the calibration system.

To obtain the measurement accuracy of the calibration system, a calibrated laser gyroscope was installed on the upper platform as the test load. The test results from the laser gyroscope serve as the reference values and are compared with the measured values from the calibration system. The angular resolution of the laser gyroscope (3σ, 10 kHz, 0.01–500 Hz) is 0.0000180″. The overall composition of the calibration system and the connections between its components are illustrated in the Figure 5.

### 3.1. Testing Methodology

The basic characteristic tests include fundamental frequency testing and accuracy testing. The fundamental frequency test is used to determine the natural vibration frequency of the vibration table and understand its inherent vibration characteristics. This can be achieved by lightly pushing the upper platform and releasing it to allow it to vibrate freely, followed by data collection and processing. The accuracy test aims to verify the reliable amplitude range of the system, ensuring stable and efficient vibration performance. This is carried out by connecting the motor to the power supply via an electrical interface, controlling the frequency and amplitude of the power signal to produce corresponding periodic torque, thereby inducing angular vibrations of specific frequencies and amplitudes on the upper platform.

During the testing process, the laser vibrometer measures the displacement and velocity of the laser spot on the target surface. Through the geometric relationships of the system, these data can be converted into the angular displacement and angular velocity of the upper platform. Let the micro-rotation angle of the upper platform be Δθ, and the displacement of the laser measurement point be Δx. The relationship between them is given by

$$\Delta x=d\sin\Delta \theta -l\left(1-\cos\Delta \theta \right)$$

where *d* is the distance between the laser measurement point and the center of the turntable, and *l* is the perpendicular distance between the laser measurement point and the target surface. In practical use, the distance from the measurement point to the center of the turntable is determined by measuring the distance from the laser point to the edge of the upper turntable using a caliper; since the upper turntable is large, it can be assumed that the measurement error has a minimal impact on the accuracy of the turntable system.

### 3.2. Testing Results

- Fundamental frequency test

**Figure 6:**
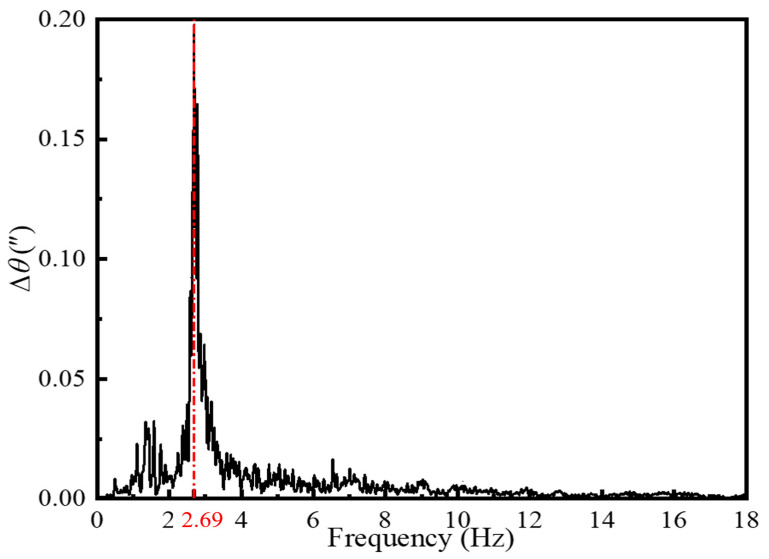
Fundamental frequency testing results.

In the fundamental frequency test, the sensitivity of the laser vibrometer, the data acquisition system, and the voltage range were adjusted accordingly. Data collection was started, excitation was applied to the turntable, and after the system stabilized, the single-frequency point characteristics of the spectrum were observed and recorded. The Fast Fourier Transform (FFT) of fundamental frequency testing results, shown in the Figure 6 below, indicate that the fundamental frequency is 2.69 Hz. During the simulation, the connections between various parts were modeled using RBE2-type MPC constraints, which are idealized constraints. However, in reality, the turntable is a structure with many assembly joints, and these connections are not as ideal as in the simulation. This discrepancy led to the measured frequencies being lower than the simulation results.

- Accuracy testing

The measurement results of angular displacement for the angular vibration table under different excitation frequencies, obtained using the designed calibration system and compared with those from the laser gyroscope, are listed in the Table 4. Compared to the laser gyroscope, the maximum measurement error is 0.00172″.

## 4. Conclusions

This study conducted a comprehensive investigation into the design and validation of a high-precision angular vibration platform. The main conclusions are as follows:

(1). A high-precision angular vibration platform with self-driving functionality was designed and implemented. The platform consists of an upper platform, a lower platform, a motor, and supporting spring plates, capable of providing precise angular vibration inputs. The design effectively addresses the need for vibration control by providing support stiffness through the spring plates while maintaining low rotational stiffness around the *z*-axis to ensure platform stability and accuracy.

(2). Modal analysis and spring plate stability analysis of the platform were conducted using ANSYS finite element analysis software. The simulation results indicate that the designed turntable has good dynamic characteristics, meeting the requirements for natural vibration frequency and stability. Additionally, the design of the spring plates ensures that the turntable remains free from excessive deformation or instability during angular vibration. The fundamental frequency test results show that the platform’s fundamental frequency is 2.69 Hz, which meets the design requirements.

(3). A measurement accuracy verification test for the calibration system was designed and implemented. Test results demonstrate that, compared to the measurements from the laser gyroscope, the maximum measurement error of the high-precision angular vibration calibration system is 0.00172″. This error falls within an acceptable range, validating the accuracy and reliability of the calibration system.

## Figures and Tables

**Table 1:** The first six modal frequencies of the upper platform.

Modal Order	Modal Frequency (Hz)
1	931.25
2	1077.2
3	1475.4
4	1882.8
5	1976.8
6	1987.9

**Table 2:** The critical stress and maximum stress of the spring plates.

Thickness (mm)	0.6	0.8	1	1.5	2	2.5	3	3.5
Critical stress (MPa)	50.7	90.1	140.8	316.8	563.3	880.1	1267.4	1725
Maximum stress (MPa)	24.8	18.6	14.9	9.91	7.43	5.94	4.95	4.25

**Table 3:** The first four modal frequencies of the overall structure.

Thickness (mm)	0.6	0.8	1	1.5	2	2.5	3	3.5
1st mode	3.9	4.73	5.59	8	10.79	13.941	17.42	21.19
2nd mode	100.94	116.54	130.28	159.32	184.17	205.89	225.55	243.64
3rd mode	100.99	116.6	130.34	159.6	184.26	205.99	225.66	243.76
4th mode	183.44	244.06	290.33	355.55	410.51	458.92	502.68	542.9

**Table 4:** Measured angular vibration values vs. reference values.

Excitation Frequency (Hz)	20	40	60	120	160
Measured value (″)	0.2044	0.1853	0.1586	0.04789	0.0318
Reference value (″)	0.2043	0.1865	0.1583	0.04617	0.0315
Relative error (″)	0.0001	0.0012	0.0003	0.00172	0.0003

## Data Availability

The original contributions presented in the study are included in the article; further inquiries can be directed to the corresponding author.
